# Alopecia Areata and malignancies: uncertainties clarified by a large-scale population-based study

**DOI:** 10.1007/s00403-024-03385-3

**Published:** 2024-10-14

**Authors:** Khalaf Kridin, Rimma Laufer-Britva, Francisco Jimenez, Arnon D. Cohen, Baruch Kaplan, Anna Lyakhovitsky

**Affiliations:** 1 Unit of Dermatology and Skin Research Laboratory, Galilee Medical Center, Nahariya, Israel; 2 Azrieli Faculty of Medicine, Bar-Ilan University, Safed, Israel; 3 Lübeck Institute of Experimental Dermatology, University of Lübeck, Lübeck, Germany; 4 Mediteknia Dermatology and Hair Transplant Clinic, Gran Canaria, Spain; 5 Ciencias de la Salud, Universidad Fernando Pessoa Canarias, Las Palmas, Spain; 6 Clínica Dermatológica Internacional/Ruber Internacional, Madrid, Spain; 7 Clalit Health Services, Tel-Aviv, Israel; 8 Faculty of Health Sciences, Ben-Gurion University of the Negev, Ben-Gurion Ave, Beer Sheva, Israel; 9 Adelson School of Medicine, Ariel University, Ariel, Israel; 10 Department of Dermatology, Sheba Medical Center, Ramat Gan, Israel; 11 Sackler Faculty of Medicine, Tel Aviv University, Tel Aviv, Israel

**Keywords:** Alopecia areata, Malignancies, Cross-sectional study, Epidemiology

## Abstract

The association of AA with malignancies has been a scope of controversy as the current literature is highly inconsistent in this regard. To evaluate the association between AA and hematological malignancies (HMs) and solid malignancies (SMs) using a large-scale, real-life computerized database. A cross-sectional study was conducted to compare the prevalence of HMs and SMs among patients with AA relative to age-, sex-, and ethnicity-matched control subjects. Chi-square and t-tests were used for univariate analysis, and a logistic regression model was used for multivariate analysis. The study included 51,561 patients with AA and 51,410 controls. AA was significantly associated with HMs (adjusted OR, 1.27; 95% CI, 1.07–1.51; *P* = 0.006). This association was more robust among patients with late-onset AA (≥ 50 years; OR, 1.33; 95% CI, 1.04–1.71; *P* = 0.025). On the other hand, AA was not found to be significantly associated with SM (adjusted OR, 0.97; 95% CI, 0.88–1.06; *P* = 0.487), excluding among patients with alopecia totalis and universalis (OR, 2.10; 95% CI, 1.03–4.27; *P* = 0.036). In a granular analysis including 5 HMs and 18 SMs, non-Hodgkin lymphoma was the only malignancy that proved positively associated with AA (adjusted OR, 1.32; 95% CI, 1.03–1.69; *P* = 0.028). AA is associated with HMs but not SMs. Further research is warranted to validate our observations in other study cohorts.

## Introduction

Alopecia areata (AA) is a prevalent immuno-inflammatory disease, driven by cytotoxic T lymphocytes directed against the anagen hair follicles, leading to nonscarring hair loss [1–3]. AA affects approximately 2% of the global population [1–3]. While AA primarily impacts the hair follicles, its association with different systemic diseases has garnered increasing attention in recent years. Higher rates of atopy, systemic lupus erythematosus, rheumatoid arthritis, vitiligo, thyroid disorders, and psoriasis have been reported among AA patients [4–8]. Alongside the exploration of the association of other autoimmune inflammatory disorders with malignancies, possibly triggered by immune dysregulation and chronic inflammation, the potential link between AA and malignancy, including both hematological malignancies (HMs) and solid malignancies (SMs), has emerged as a significant area of interest [9–13].

Several studies have pointed out a potential association between AA and cancer, yielding conflicting results [8–12, 14–19]. A nationwide population-based matched cohort study conducted in Taiwan reported a decreased overall cancer risk in AA patients, especially among males. However, AA was associated with an increased risk of lymphoma, breast, kidney, and bladder cancers in females and a decreased risk of non-melanoma skin cancer (NMSC), upper gastrointestinal, liver, uterine, and cervical cancers [9]. Two studies from Korea evaluated the malignancy risk in AA patients. One study found an increased overall cancer risk and differences between patients with alopecia totalis (AT) and alopecia universalis (AU) compared to other AA patients, with a higher risk of thyroid cancer in those with AT or AU, and a higher risk of prostate and bladder cancer in non-AU/AT AA patients [11]. Another study found an increased risk of thyroid cancer but a decreased risk of breast, colon/rectum, stomach, lung, and liver cancer in AA patients [13]. A claims-based study conducted in the US did not reveal an increase in malignancy rates [10]. Mostaghimi et al. revealed a decrease in NMSC among AA patients [12]. Several case reports have documented an association between AA and HMs [14–19]. Additionally, prior research suggests different comorbidity profiles in late-onset AA, defined by several researches as AA with onset after 50 years [20–22]. One study indicates a possibility of an increased risk of malignancy in this age group of AA patients [20].

Given the mixed findings and limited large-scale studies, further investigation is warranted to clarify the potential link between AA and malignancies. The primary goal of this study was to elucidate the association between AA and various HMs and SMs using a large, population-based dataset. The secondary goals were to evaluate the malignancy risk in late-onset AA and in patients with AT and AU compared to other AA patients.

## Methods

### Study design and dataset

The current study was performed to investigate the association of AA with a wide array of HMs and SMs using a large population-based study. A retrospective cross-sectional study was conducted based on the computerized database of Clalit Health Services (CHS). CHS is the largest healthcare maintenance organization in Israel, providing a wide array of private and public healthcare services for 4,927,000 enrollees as of October 2018. The different characteristics of the utilized dataset are detailed in other publications [23, 24].

### Study population and definition of the main variables

The dataset of CHS was systematically checked for incident cases with a diagnostic code AA between the years 2002 and 2019. Patients were eventually defined as eligible for inclusion only if one of the following criteria was met: (i) a documented diagnosis of AA registered at least twice by a board-certified dermatologist, or (ii) diagnosis of AA in discharge letters of patients admitted to dermatological wards. We additionally recruited a control group, including one enrollee lacking a diagnosis of AA per each case of AA. Controls were matched based on sex, age, and ethnicity.

The diagnosis of each of the HMs and SMs was based on its documentation in the cancer registry of the CHS. This registry is cross-linked with the National Cancer Registry and undergoes continuous updates and logarithmic checks [24]. The HM variable was defined as the occurrence of one of the following 5 diseases: acute leukemia, chronic leukemia, Hodgkin lymphoma, non-Hodgkin lymphoma, and multiple myeloma. Definition of the SM variable relied on the registration of any of the following cancers: breast, bone, brain, cervix, colorectal, esophagus, kidney, larynx, liver and bile ducts, lung, ovary, pancreas, pharynx, prostate, stomach, thyroid, uterus, and urinary bladder. In cases where individuals had more than one type of HM or SM, the date of the initial disease diagnosis was utilized to calculate the index date. Our analyses were run on the pooled HM and SM variables and on each one of their 5 and 18 types, respectively.

Outcome measures were controlled for the Charlson comorbidity index, a well-established epidemiological tool utilized to evaluate the severity and significance of concurrent medical conditions. This index is frequently used in epidemiological studies and has displayed strong reliability in forecasting mortality [25]. To eliminate bias, we used a modified version of the score following the exclusion of the malignancy component of the scoring system. In addition, outcome measures were additionally adjusted for age, sex, ethnicity, socioeconomic status (SES), and body mass index (BMI). Two sensitivity analyses were performed to elucidate the association in specific subgroups. The first concentrated on patients with AT and AU (*n* = 198), and the second on patients who presented with AA after the age of 50 years (late-onset AA) [20].

### Statistical analysis

Baseline characteristics were described by means and standard deviations (SD)s for continuous variables, while categorical values were signified by percentages. To compare sociodemographic and clinical factors between cases and controls, we employed the Chi-square test for categorical variables and the t-test for continuous variables.

We utilized logistic regression to determine odds ratios (ORs) and their corresponding 95% confidence intervals (CIs). This association was computed based on individuals who developed PG after the diagnosis of each specific HM, taking into account the temporal relationship between exposure and outcome in case-control studies. Statistical significance was defined as two-tailed P-values below 0.05. All statistical analyses were performed using SPSS software, version 25 (SPSS, Armonk, NY: IBM Corp).

## Results

### Characteristics of the study population

Our study population comprised 51,561 patients with AA and 51,410 age-, sex-, and ethnicity-matched control subjects. The mean (SD) age of patients with AA was 34.1 (17.1), which is identical to the age of control subjects at their enrollment date. In all, 31,085 (60.3%) of AA patients were female, and 67.5% were of Jewish ancestry. Comorbidity rates, measured by the Charlson comorbidity index, were comparable between the two groups, with 1,352 (2.6%) AA patients and 1,354 (2.6%) control subjects having severe comorbidities (Table 1).

**Table 1:** Descriptive characteristics of the study population

Characteristic	Patients with alopecia areata (*N* = 51,561)	Controls (*N* = 51,410)	*P* value
Age, years			
Mean (SD)	34.1 (17.1)	34.1 (17.0)	1.000
Median (range)	34.0 (0–99.0)	34.0 (0–99.0)	
Male sex, N (%)	31,085 (60.3%)	31,001 (60.3%)	1.000
Ethnicity, N (%)			
Jews	34,800 (67.5%)	34,656 (67.4%)	0.732
Arabs	16,761 (32.5%)	16,754 (32.6%)	
SES, N (%)			
Low	25,218 (48.9%)	25,179 (48.9%)	1.000
Intermediate	17,396 (33.7%)	17,328 (33.7%)	
High	8,758 (17.0%)	8,720 (17.0%)	
Unknown	189 (0.4%)	183 (0.4%)	
Charlson comorbidity score, n (%)			
None (0)	42,076 (81.6%)	42,383 (82.4%)	**< 0.001**
Moderate (1–2)	8,133 (15.8%)	7,673 (14.9%)	**< 0.001**
Severe (≥ 3)	1,352 (2.6%)	1,354 (2.6%)	1.000
Body mass index (Kg/m2), Mean (SD)	25.5 (6.3)	25.9 (5.6)	**< 0.001**

*Abbreviations* N, Number; SD, standard deviation; BMI, body mass index; SES, socioeconomic status

### The association of AA with HMs

Table 2 demonstrates the association of AA with HMs. The prevalence of HM was greater in patients with AA than in control (0.6% vs. 0.5%, respectively; *P* = 0.006). Thus, AA was significantly associated with HM (OR, 1.27; 95% CI, 1.07–1.51). In an age-, sex-, and ethnicity-stratified analysis, the association was prominently heightened among older individuals (≥ 34 years; OR, 1.28; 95% CI, 1.06–1.55; *P* = 0.011) males (OR, 1.34; 95% CI, 1.08–1.66; *P* = 0.008), and Jews (OR, 1.27; 95% CI, 1.05–1.54; *P* = 0.014).

**Table 2:** The association between Alopecia Areata and hematologic malignancies stratified by age, gender, and ethnicity

Subgroup		HM in patients with alopecia areata (*N* = 51,561); *n* (%)	HM in controls (*N* = 51,410); *n* (%)	OR (95%CI)	*P* value
**All**		299 (0.6%)	235 (0.5%)	**1.27 (1.07–1.51)**	**0.006**
**Age**,** years**					
< 34		58 (0.2%)	47 (0.2%)	1.23 (0.84–1.81)	0.287
≥ 34		241 (0.9%)	188 (0.7%)	**1.28 (1.06–1.55)**	**0.011**
**Gender**					
Male		193 (0.6%)	144 (0.5%)	**1.34 (1.08–1.66)**	**0.008**
Female		106 (0.5%)	91 (0.4%)	1.16 (0.88–1.54)	0.295
**Ethnicity**					
Jews		242 (0.7%)	190 (0.5%)	**1.27 (1.05–1.54)**	**0.014**
Arabs		57 (0.3%)	45 (0.3%)	1.27 (0.86–1.87)	0.235
**Sensitivity analyses**					
**AA totalis or AA universalis (** *N* ** = 198)**					
		0 (0.0%)	235 (0.5%)	NA	NA
**Late-onset AA (≥ 50 years)**					
		144 (1.6%)	108 (1.2%)	**1.33 (1.04–1.71)**	**0.025**

*Abbreviation* N, Number; HM, hematologic malignancies; AA, alopecia areata; NA, not applicable; OR, odds ratio; CI, confidence interval

To assess whether there is an independent association between the conditions, we performed a multivariate logistic regression analysis, which controlled for demographic factors and comorbidities. Consistent with the findings from the univariate analysis, HMs were found to be independently associated with AA (adjusted OR, 1.27; 95% CI, 1.07–1.51; *P* = 0.006).

We then carried out a sensitivity analysis shedding light on patients with late-onset AA (> 50 years). Similarly, a statistically significant association was found between AA and HM (OR, 1.33; 95% CI, 1.04–1.71; *P* = 0.025).

### The association of AA with SMs

Analysis of the association of AA with SMs is depicted in Table 3. The prevalence of SM was comparable between patients with AA and control subjects (1.9% vs. 2.0%, respectively), signifying that no significant association was found between AA and SM (OR, 0.97; 95% CI, 0.89–1.06; *P* = 0.543). The lack of statistical significance persisted in age-, sex-, and ethnicity-stratified analyses (Table 3).

**Table 3:** The association between Alopecia Areata and solid malignancies stratified by age, gender, and ethnicity

Subgroup		SM in patients with alopecia areata (*N* = 51,561); *n* (%)	SM in controls (*N* = 51,410); *n* (%)	OR (95%CI)	*P* value
**All**		986 (1.9%)	1010 (2.0%)	0.97 (0.89–1.06)	0.543
**Age**,** years**					
< 34		71 (0.3%)	68 (0.3%)	1.04 (0.75–1.45)	0.808
≥ 34		915 (3.5%)	942 (3.6%)	0.97 (0.88–1.06)	0.470
**Gender**					
Male		337 (1.1%)	366 (1.2%)	0.92 (0.79–1.07)	0.256
Female		649 (3.2%)	644 (3.2%)	1.01 (0.89–1.12)	0.935
**Ethnicity**					
Jews		867 (2.5%)	876 (2.5%)	0.99 (0.89–1.08)	0.760
Arabs		5 (0.0%)	3 (0.0%)	0.89 (0.69–1.14)	0.342
**Sensitivity analyses**					
**AA totalis or AA universalis (** *N* ** = 198)**					
		8 (4.0%)	1010 (2.0%)	**2.10 (1.03–4.27)**	**0.036**
**Late-onset AA (≥ 50 years)**					
		710 (7.9%)	718 (8.0%)	0.98 (0.88–1.09)	0.735

*Abbreviations* N, Number; SM, solid malignancies; AA, alopecia areata; NA, not applicable; OR, odds ratio; CI, confidence interval

After controlling for putative confounding factors, AA demonstrated no independent association with SM in multivariable logistic regression analysis (adjusted OR, 0.97; 95% CI, 0.88–1.06; *P* = 0.487). Interestingly, in a sensitivity analysis confined to patients with AT and AU and their control counterparts, SM was found to be significantly associated (OR, 2.10; 95% CI, 1.03–4.27; *P* = 0.036).

### Association between AA and different types of HMs and SMs

We then performed a granular analysis investigating the association of AA with 5 different types of HMs and 18 different types of SMs (Table 4). AA displayed an independently significant association with non-Hodgkin lymphoma (adjusted OR, 1.32; 95% CI, 1.03–1.69; *P* = 0.028). On the other hand, an inverse association was revealed between AA and uterus cancer (adjusted OR, 0.56; 95% CI, 0.33–0.95; *P* = 0.032). None of the remaining types of SM and HM was found to be significantly associated with AA (Table 4).

**Table 4:** The association between Alopecia Areata and different hematologic and solid malignancies

	*N* of cases (among AA, among controls)	OR (95%CI)	*P* value	Adjusted OR (95%CI)^a^	*P* value
**Hematologic malignancies**					
Acute leukemia	(34, 37)	0.92 (0.58–1.46)	0.712	0.94 (0.57–1.54)	0.810
Chronic leukemia	(37, 28)	1.32 (0.81–2.15)	0.269	1.35 (0.81–2.24)	0.247
Hodgkin lymphoma	(66, 52)	1.27 (0.88–1.82)	0.203	1.29 (0.88–1.89)	0.187
Non-Hodgkin lymphoma	(159, 115)	**1.38 (1.09–1.75)**	**0.008**	**1.32 (1.03–1.69)**	**0.028**
Multiple myeloma	(20, 17)	1.17 (0.61–2.24)	0.628	1.16 (0.60–2.21)	0.663
**Solid malignancies**					
Breast cancer^b^	(354, 349)	1.01 (0.87–1.17)	0.881	1.01 (0.87–1.16)	0.921
Bone cancer	(14, 15)	0.93 (0.45–1.93)	0.846	1.14 (0.53–2.47)	0.739
Brain and CNS cancer	(30, 22)	1.36 (0.78–2.36)	0.272	1.62 (0.89–2.95)	0.118
Cervix cancer^b^	(25, 27)	0.92 (0.54–1.59)	0.773	0.97 (0.56–1.69)	0.917
Colorectal cancer	(90, 112)	0.80 (0.61–1.06)	0.116	0.79 (0.60–1.05)	0.098
Esophagus cancer	(1, 0)	NA	0.318	NA	0.953
Kidney cancer	(41, 41)	0.99 (0.65–1.54)	0.989	0.98 (0.63–1.51)	0.919
Larynx cancer	(16, 15)	1.06 (0.53–2.15)	0.864	1.01 (0.50–2.04)	0.989
Liver and bile duct cancer	(6, 1)	5.98 (0.72–49.69)	0.059	5.44 (0.65–45.69)	0.119
Lung cancer	(41, 41)	0.99 (0.65–1.54)	0.989	0.94 (0.60–1.45)	0.761
Ovary cancer	(22, 23)	0.95 (0.53–1.71)	0.874	0.93 (0.51–1.69)	0.814
Pancreas cancer	(9, 6)	1.50 (0.53–4.20)	0.442	1.37 (0.49–3.86)	0.552
Pharynx cancer	(25, 21)	1.19 (0.66–2.12)	0.562	1.29 (0.71–2.35)	0.401
Prostate cancer^c^	(59, 51)	1.15 (0.79–1.68)	0.455	NA	0.552
Stomach cancer	(18, 14)	1.28 (0.64–2.58)	0.485	1.25 (0.62–2.52)	0.534
Thyroid cancer	(95, 92)	1.03 (0.77–1.37)	0.842	1.03 (0.77–1.37)	0.869
Uterus cancer^b^	(22, 37)	**0.59 (0.35-1.00)**	**0.049**	**0.56 (0.33–0.95)**	**0.032**
Urinary bladder cancer	(58, 56)	1.03 (0.72–1.49)	0.864	1.20 (0.87–1.68)	0.269

*Abbreviations* AA, alopecia areata; n, Number; OR, odds ratio; CI, confidence interval; CNS, central nervous system; NA, not applicable

^a^-Multivariate logistic regression model adjusting for age, sex, ethnicity, and comorbidities

^b^- Calculated among females

^c^-Calculated among males

## Discussion

The present study aimed to elucidate the association between AA and various malignancies, including HMs and SMs, using a large, population-based dataset. Additionally, it evaluates the malignancy risk in late-onset AA compared to other AA patients and the malignancy risk in AT and AU patients compared to other AA patients. The findings offer new insights and highlight the need for further research in this area. Relative to controls, we found that the lifetime prevalence of HMs was higher among patients with AA, particularly among patients with late-onset AA. The lifetime prevalence of SM was comparable between patients with AA and controls, except those with AT and AU, who exhibited a significant association with SMs. In a granular analysis, non-Hodgkin lymphoma was the only malignancy that was positively and significantly associated with AA.

### Association of AA with HMs

Our results indicate a significant association between AA and HMs, with AA patients exhibiting a higher prevalence of HMs compared to controls. Specifically, the odds of developing HMs were 1.27 times higher in AA patients, with non-Hodgkin lymphoma being particularly associated with AA. These findings align with a previous study and case reports that have reported a link between AA and HMs [9, 14–19]. The heightened risk among older individuals, males, and those of Jewish ancestry further underscores the potential demographic variations in this association. The sensitivity analysis focusing on late-onset AA patients also demonstrated a statistically significant association, suggesting that age of onset may influence the malignancy risk in AA patients. This finding is particularly relevant given the different comorbidity profiles observed in late-onset AA, as highlighted in prior research [20–22]. The significant association between AA and HMs found in our study suggests considering the need for monitoring HMs in AA patients, particularly those with late-onset AA.

### Association of AA with SMs

In contrast to the findings with HMs, the analysis did not reveal a significant association between AA and SMs overall. The prevalence of SMs was comparable between AA patients and controls, and no independent association was observed after adjusting for confounding factors. This is concordant with several previous studies and different from others [9–11]. The lack of a significant association with SMs overall suggests that AA may not impose a broad risk factor for solid tumors. However, the sensitivity analysis confined to AT and AU patients revealed a significant association with SMs, suggesting that the severity of AA may play a role in cancer risk. This indicates that these subgroups of AA patients may still be at increased risk and should be monitored accordingly. Interestingly, our results revealed an inverse association between AA and uterine cancer, indicating a potential protective effect. To note, one prior study also found differences in types of malignancies in AT and AU patients compared to other AA patients, revealing increased thyroid cancer in AT and AU, and increased prostate and bladder cancer in other AA patients [11]. The differences in cancer rates may be influenced by geographic variations, warranting further evaluation to understand the underlying mechanisms and potential clinical implications.

### Strengths and limitations

Despite the strengths of the large, population-based dataset from all healthcare system tiers, matched control groups, diagnoses of AA made by certified dermatologists, and robust statistical analyses, the study has several limitations. Since our data was obtained from computerized, anonymized medical records and relied on diagnostic codes, it may result in misclassification or underreporting of AA and malignancy cases. Additionally, the study population was limited to a specific geographic region, which may limit the generalizability of the findings to other populations. Conducting longitudinal multi-center studies across diverse populations can help identify regional and ethnic variations in the association between AA and malignancies. This can lead to more culturally and regionally tailored healthcare strategies and improve the generalizability of findings.

In conclusion, this study provides evidence of a significant association between AA and HMs, particularly non-Hodgkin lymphoma, highlighting the increased association in late-onset AA and male patients. Although there was no overall increase in SMs for all AA patients, it was higher in those with AU and AT. The study underscores the importance of considering demographic factors such as age, sex, ethnicity, and disease severity when assessing cancer risk in AA patients. Future research should focus on confirming these associations, elucidating the underlying mechanisms, and developing preventive strategies to improve clinical management.

## Data Availability

No datasets were generated or analysed during the current study.
